# Long intervals between repetitive concussions reduce risk of cognitive impairment and limit microglial activation, astrogliosis, and tauopathy in adolescent rats

**DOI:** 10.1038/s41598-025-24376-y

**Published:** 2025-11-18

**Authors:** Yuichi Hirata, Kyohei Kin, Takayuki Nagase, Tatsuya Sasaki, Susumu Sasada, Chiaki Sugahara, Takahiro Hirayama, Koji Kawai, Shun Tanimoto, Hayato Miyake, Tomoya Saijo, Hiromichi Naito, Kaori Masai, Takao Yasuhara, Shota Tanaka

**Affiliations:** 1 Department of Neurological Surgery, Okayama University Graduate School of Medicine, Dentistry and Pharmaceutical Sciences, Okayama University Hospital, 2-5-1, Shikata-cho, Kita-ku, Okayama, 700-8558 Japan; 2 Department of Emergency, Critical Care, and Disaster Medicine, Okayama University Graduate School of Medicine, Dentistry, and Pharmaceutical Sciences, Okayama University Hospital, 2-5-1, Shikata-cho, Kita-ku, Okayama, 700-8558 Japan; 3 Department of Medical Neurobiology, Okayama University Graduate School of Medicine, Dentistry and Pharmaceutical Sciences, 2-5-1, Shikata-cho, Kita-ku, Okayama, 700-8558 Japan; 4 Yasuhara Clinic, 979 Kubaracho, Marugame, 763-0073 Japan

**Keywords:** Concussion, Return to play, Sports-related head injury, Microglia, Astrocyte, Tauopathy, Neuroscience, Diseases, Neurology

## Abstract

Although previous studies have demonstrated the effects of concussions do not accumulate as the time interval between injuries increases, little is known about the relationship between this interval and the effects of repetitive concussions. The objective of this study is to explore the relationship between the time interval and changes in behavior and histology following repetitive concussions. Male adolescent rats received concussions by weight drop and were randomly assigned to one of five experimental groups, receiving concussions three times either daily, every other day, once per week, once every 2 weeks, or receiving sham procedures. Only rats that received daily concussions exhibited cognitive impairment, while the other groups did not. No groups showed motor or anxiety-like impairments. Histological analysis revealed accumulation of microglia, as well as astrogliosis, in the prefrontal cortex, corpus callosum, dentate gyrus, and cornu Ammonis 1 region of the hippocampus in rats subjected to daily concussions. Accumulation of phosphorylated tau was also observed in the prefrontal cortex and cornu Ammonis 1. Longer intervals between concussions may reduce the risk of cognitive impairment and limit microglial activation, astrogliosis, and phosphorylated tau accumulation. These findings may help guide decisions on the appropriate timing for return to play in humans.

**Supplementary Information:**

The online version contains supplementary material available at 10.1038/s41598-025-24376-y.

## Introduction

Concussion results from head trauma, and can sometimes lead to persistent and progressive motor or cognitive impairment^1,2^. According to the 2017 Centers for Disease Control and Prevention report, the overall incidence of traumatic brain injury (TBI) has increased from 534.4 to 787.1 per 100,000 since 2007^3^. The majority of TBIs are considered mild TBI (mTBI), with peak incidence among adolescents and young adults. Injuries frequently occur during sports or recreational activities^3,4^. Helmets and mouthguards are used for protection, but they do not provide complete defense against concussion^5^. Cognitive impairment following a concussion is known to emerge soon after the injury and typically recovers spontaneously in most cases^6^. However, in some instances, concussion can lead to lasting motor or cognitive impairment. In addition, sustaining another head injury during the vulnerable time window following concussion can result in complications such as cerebral edema and neurological deterioration^7–9^. This highlights the importance of adequate recovery time, and longer recovery durations have been associated with better cognitive activity in humans^10,11^. Repetitive concussions may also lead to chronic traumatic encephalopathy (CTE), which presents with cognitive, behavioral, and emotional impairments decades later^12^. Determining when to return to sport after a concussion remains a challenge. Several tools are currently used to guide return to play decisions^13–16^. While these guidelines generally recommend rest after concussion, the effectiveness of rest remains unclear in humans, making it difficult to define the optimal duration of time away from play. Concussion causes ionic dyshomeostasis, excitatory amino acid release, and alterations in cerebral blood flow. These changes may increase vulnerability to subsequent concussions^17,18^. Animal studies have demonstrated that cognitive impairment persists when concussions are repeated within the vulnerable time window, whereas sufficient intervals between injuries can prevent long-term cognitive impairment. Notably, short-interval concussions are associated with prolonged cognitive impairment and microglial activation^19,20^. Microglial activation and phosphorylated tau accumulation have both been associated with cognitive impairment^21,22^. These findings suggest that extending the time interval between concussions may be essential in reducing the risk of long-term cognitive impairment. While the cumulative effects of concussions may be mitigated by longer intervals, little is known about the precise relationship between time intervals and the effects of repetitive concussions. The objective of this study is to explore the relationship between the time interval and changes in behavior and histology following repetitive concussions.

## Material and methods

### Animals

For all experiments, male Wistar rats (5 weeks of age at the start of the study; Jackson Laboratory Japan, Inc., Yokohama, Japan) were utilized. The animals were housed in a controlled facility with a 12-h light/dark cycle and had free access to food and water. Euthanasia was performed via intraperitoneal injection of a mixed solution containing 0.3 mg/kg of medetomidine, 4.0 mg/kg of midazolam, and 5.0 mg/kg of butorphanol. All efforts were made to minimize animals’ distress throughout the study.

### Ethical statement

All experiments were conducted in accordance with the guidelines of the Institutional Animal Care and Use Committee (IACUC) of Okayama University, and reported in compliance with the Animal Research: Reporting in Vivo Experiments (ARRIVE) guidelines. The study protocol received specific approval from the IACUC of Okayama University (Approval Number: OKU-2024477).

### Experimental design

**Fig. 1:**
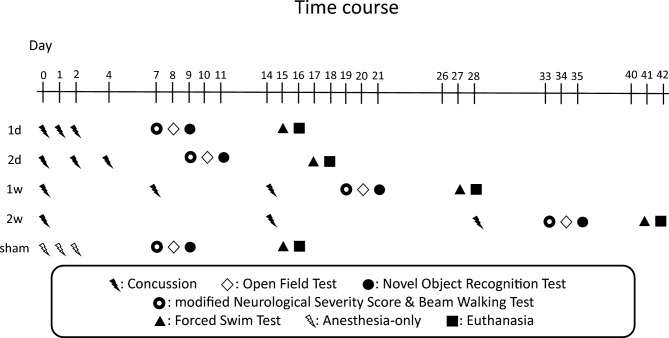
Time course. Experimental designs show the timing of concussion and behavioral assessment (n = 8 in each group). In all groups, the first concussion was induced on day 0. The timing of behavioral assessment and euthanasia are illustrated in the schematic.

Animals were randomly assigned to one of five experimental groups: the 1 d group, 2 d group, 1w group, 2w group, and sham group (n = 8/group). Rats in the 1 d, 2 d,1w, and 2w groups received three concussions in total, administered either every day, every other day, once per week, or once every 2 weeks, respectively. Rats in the sham group received anesthesia only, on the same days that the 1 d group received concussions. The timeline of the experimental course is shown in Fig. 1.

### Concussion induction

Concussion was induced using a custom-made weight-drop device reported by Sugahara et al^21^. This device was employed to model concussions in rats. A 52 g ball was dropped from a height of 30 cm to induce a concussion without causing intracranial hemorrhage. Prior to the weight drop, rats were anesthetized with 5.0% isoflurane (4 l/min oxygen flow rate) and placed on the apparatus. A plastic disc (10 mm in diameter and 2 mm thick) was affixed to the midline, aligned with the bregma. Impact force was measured using a designated sensor (Valcom Co., Ltd., Osaka, Japan), which can evaluate impact forces ranging from 0.001 to 49 N. Following the concussion, rats were placed in a clean cage for recovery in room air. Rats in the 1 d group received concussions on day 0, 1, and 2. Rats in the 2 d group received concussions on day 0, 2, and 4. Rats in the 1w group received concussions on day 0, 7, and 14. Rats in the 2w group received concussions on day 0, 14, and 28.

### Behavioral assessments

The modified neurological severity score (mNSS) and beam walking test (BWT) were used to assess motor impairment. Cognitive impairment and/or depression-like behaviors were assessed using the open field test (OFT), novel object recognition test (NORT), and forced swim test (FST). All groups underwent behavioral assessments in the following sequence after the final concussion: mNSS and BWT on day 5, OFT on day 6, NORT on day 7, and pre-FST and FST on day 12 and 13, respectively. All rats were euthanized using institution-approved methods and subsequently sacrificed for immunohistological evaluation on day 14 after the final concussion.

The NORT was performed to assess cognitive function following concussion, as described in a previous report^23^. It was performed the day after the OFT in the same arena to ensure habituation during the OFT. The testing arena measured 100 cm in width, 100 cm in depth, and 80 cm in height. During the OFT, rats were given 10 min to freely explore the arena. During the NORT, two identical objects were placed at opposite sides of the arena, and rats were allowed to explore freely for 5 min. After a 60-min interval, one object was replaced with a novel object, and rats explored the objects again for 5 min during the test phase. The proportion of time spent exploring the novel object (time spent with the novel object/total object exploration time × 100) was calculated. These proportions were compared across all groups. A preference for the novel object over the familiar one reflects intact recognition memory. The arena was cleaned with 70% ethanol before each trial.

To assess neurological function, the mNSS and BWT were performed. The mNSS evaluates motor, sensory, reflex, and balance functions. Scores range from 0 to 18, with higher scores indicating more severe neurological impairment^24,25^. The BWT evaluates motor and balance functions^26,27^. Rats were placed at the end of a 60-cm beam and allowed to walk toward their home cage at the opposite end. Beams of 3 cm, 2 cm, and 1 cm in width were used, and each rat was tested three times per beam. The number of foot slips were recorded during each trial.

The OFT was performed to assess anxiety-like behavior and locomotor activity in a novel arena, as described previously^23^. The arena was divided into nine squares, each measuring 33 cm × 33 cm. Time spent in the center of the arena was also recorded as an index of anxiety-like behavior^28,29^. Rats were placed into the arena from one corner and allowed to freely explore for 10 min. Movements were video-recorded and analyzed using Ethovision® XT 9.0 software (Noldus, Wageningen, Netherlands). The arena was cleaned with 70% ethanol before each trial. Total distance moved and time spent in the center area were measured.

The FST was performed to assess depression-like behavior, as described previously^30^. A cylinder (20 cm in diameter, 45 cm in height) was filled with water to a depth of 30 cm. Water temperature was maintained at 22 ± 2°C. On the first day, rats were placed in the water-filled cylinder for 15 min. Twenty-four hours later, they were returned to the water-filled cylinder again and tested for 5 min. All movements were video-recorded and analyzed using Ethovision® XT 9.0 software to calculate immobility time, which was used as an index of depression-like behavior.

### Histological evaluations

The free-floating method was employed for all procedures. Ionized calcium-binding adapter molecule 1 (Iba-1) and glial fibrillary acidic protein (GFAP) staining were used to assess microglial and astrocytic changes, respectively. Accumulation of phosphorylated tau (p-tau) was also observed. All staining was analyzed in the prefrontal cortex (PFC), corpus callosum (CC), dentate gyrus (DG), and cornu Ammonis 1 (CA1) region of the hippocampus.

All rats were sedated and perfused with 200 ml of ice-cold phosphate-buffered saline (PBS), followed by 200 ml of 4% paraformaldehyde (PFA) in PBS. Brains were carefully removed and post-fixed in PFA overnight at 4°C. Subsequently, brains were immersed in 30% sucrose until fully saturated. Coronal sections were cut at 30-μm intervals using a freezing microtome and stored in cryopreservation solution. Six rats from each group were used for histological analysis. For cell counting, sections from 0.7 mm anterior and 0.7 mm posterior to the bregma were taken for the PFC and CC^31^. Sections from 2.8 mm to 4.2 mm posterior to the bregma were taken for the DG and CA1 in the hippocampus^32^. In this study, staining of Iba-1, GFAP, and p-tau was performed to assess microglia, astrogliosis, and p-tau accumulation, respectively.

For Iba-1 and GFAP staining, sections were rinsed three times with PBS containing 0.1% TritonX-100 (Nacalai Tesque Inc., Kyoto, Japan). After rinsing, sections were incubated overnight at 4°C with anti-Iba-1 (1:250 rabbit, #011–27991; Fujifilm Wako, Osaka, Japan) or anti-GFAP (1:500, rabbit, NB300-141; Novus Biologicals, Littleton, CO, USA), 10% normal horse serum (Invitrogen, Carlsbad, California, USA), and 0.1% TritonX-100. The next day, sections were rinsed with PBS and incubated for 1 h at room temperature with the following secondary antibodies: anti-rabbit FITC (1:100, rabbit, cat#711-095-152: AB_2315776; Jackson Immuno Research Laboratories, Inc., West Grove, PA, USA) or anti-rabbit Cy3 (1:200, goat, ab97075; abcam, Cambridge, United Kingdom), and DAPI (4,6-diamidino-2phenylindole; 2 drops/ml, R37606; Thermo Fisher, Waltham, MA, USA) was also applied to visualize nuclei. For p-tau staining, TBS (Tris-buffered saline) and 10% normal goat serum (Invitrogen) were used. In addition, Anti-p-tau (1:500, rabbit, 44-750G, Invitrogen) and anti-rabbit Alexa Fluor 488 (1:1,000, goat, A-11008, Invitrogen) were used as the primary and secondary antibodies, respectively.

For Iba-1 and GFAP staining, the number of Iba-1 or GFAP positive cells in a 200 µm × 200 µm area was counted. For evaluation of the number of Iba-1 positive microglia exhibiting morphological features of activation, characterized by retracted processes and amoeboid morphology, semiquantitative analysis was also performed^33,34^. The number of these morphologically activated microglia in a 200 µm × 200 µm area was counted and scored as 1+, 2+, or 3+ to indicate 0–1 cells, 2–3 cells, and more than 4 cells, respectively. Measurements for each region were taken from six sections at 30-μm intervals, and the counts were averaged^35^. The density of fluorescently labeled p-tau above a threshold intensity level in the injured area was evaluated using BZ-X analyzer software (Keyence, Osaka, Japan).

### Statistical analysis

All statistical analyses were conducted using GraphPad Prism 10 (GraphPad Software, San Diego, CA, USA). Data normality was assessed using the Shapiro-Wilk test. Normally distributed data were analyzed using unpaired *t*-tests and/or one-way analysis of variance (ANOVA) followed by Bonferroni adjustments when applicable. If not normally distributed, data were analyzed using Mann-Whitney *U*-tests and/or Kruskal-Wallis tests followed by Dunn’s *post hoc* analysis when applicable. Data are presented as the mean ± standard deviation (SD) and as median values with interquartile ranges.

## Results

### Short-interval concussions aggravated cognitive function in rats

All behavioral assessments were performed according to the protocol shown in Fig. 1, and the force of each concussion is shown in Table 1. Although the euthanasia date varied among groups due to the experimental time course, there was no significant difference in weight gain between the groups up to day 14 (Supplementary Fig. S1).

**Table 1:** Impact force in each condition.

	First concussion		Second concussion		Third concussion	
	Average ± SD (N)	Median (IQR) (N)	Average ± SD (N)	Median (IQR) (N)	Average ± SD (N)	Median (IQR) (N)
1d	0.00625 ± 0.00066	0.006 (0.005 - 0.007)	0.005875 ± 0.00117	0.006 (0.004 - 0.007)	0.005625 ± 0.00122	0.0055 (0.004 - 0.007)
2d	0.0055 ± 0.00866	0.0055 (0.004 - 0.007)	0.00575 ± 0.00083	0.0055 (0.005 - 0.007)	0.0055 ± 0.00112	0.005 (0.004 - 0.008)
1w	0.00538 ± 0.00086	0.006 (0.004 - 0.006)	0.004625 ± 0.00048	0.005 (0.004 - 0.005)	0.006 ± 0.0005	0.006 (0.005 - 0.007)
2w	0.00525 ± 0.00066	0.005 (0.004 - 0.006)	0.005875 ± 0.00078	0.006 (0.004 - 0.007)	0.006 ± 0.00112	0.006 (0.004 - 0.008)

**Fig. 2:**
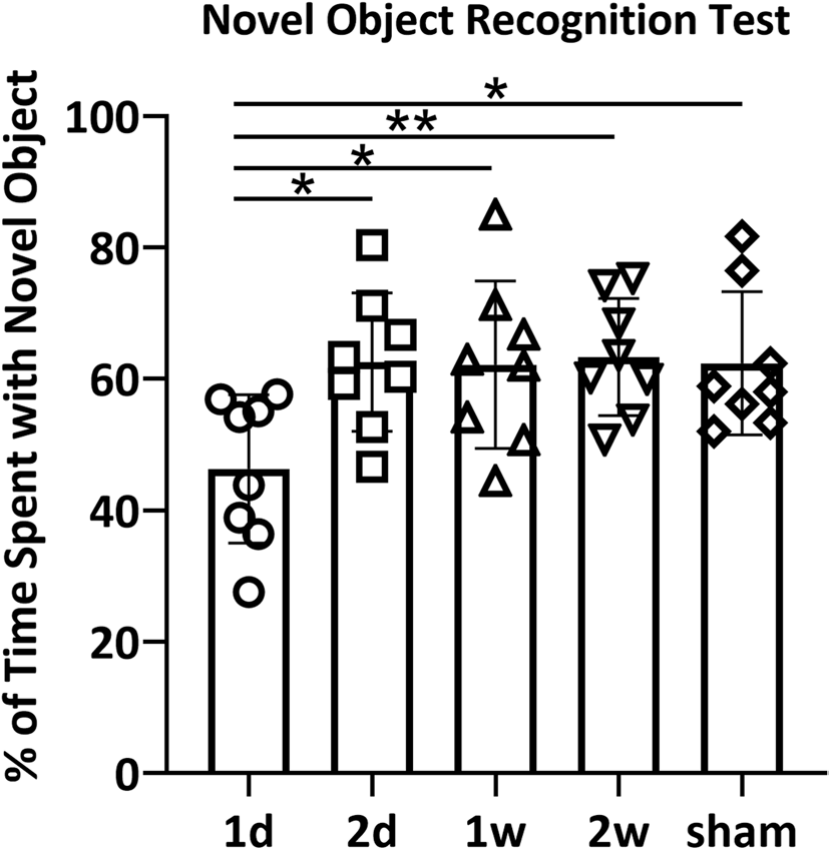
Results of the NORT. One-way ANOVA revealed a significant difference in NORT performance among groups (*p* = 0.0155, 1d: 49.0 ± 10.6%; 2d: 62.6 ± 9.8%; 1w: 62.1 ± 12.0%; 2w: 61.9 ± 8.3%; sham: 62.4 ± 10.2%). In addition, unpaired *t*-test revealed significant deterioration in the 1 d group compared to all other groups (1d vs. 2 d; *p* = 0.0100, 1 d vs. 1w; *p* = 0.0197, 1 d vs. 2w; *p* = 0.0048, 1 d vs. sham; *p* = 0.0117, respectively) (*p < 0.05, **p < 0.01).

For the NORT, one-way ANOVA revealed a significant difference among groups (*p* = 0.0155, 1d: 49.0 ± 10.6%; 2d: 62.6 ± 9.8%; 1w: 62.1 ± 12.0%; 2w: 61.9 ± 8.3%; sham: 62.4 ± 10.2%). In the 1 d group, the time spent with the novel object was significantly shorter compared to the other groups, suggesting cognitive impairment (Fig. 2).

### Effects of repetitive concussions on motor function in rats

There were no significant differences in mNSS scores among groups (*p* = 0.7811, 1d: 1^1–3^; 2d: 1^1–3^; 1w: 1 [0–3]; 2w: 2 [0–3]; sham: 1.5 [0–3]) or in the number of foot slips on the 3 cm, 2 cm, and 1 cm beams (*p* = 0.7868, *p* = 0.4638, *p* = 0.8445, respectively; 1d: 1.5 ± 1.3, 1.6 ± 1.2, 3.6 ± 2.1; 2d: 1.8 ± 0.8, 1.5 ± 1.0, 2.8 ± 1.1; 1w: 1.3 ± 0.7, 1.0 ± 0.7, 3.1 ± 1.5; 2w: 1.1 ± 1.1, 0.9 ± 0.8, 2.9 ± 1.6; sham: 1.4 ± 0.9, 1.5 ± 0.9, 3.3 ± 1.2) (Supplementary Fig. S2, 3).

### Effects of repetitive concussions on depression-like behaviors in rats

For the OFT, there were no significant differences in total distance moved (*p* = 0.6439, 1d: 42.5 [34.3–48.3.3.3] m; 2d: 49.8 [45.3–52.8.3.8] m; 1w: 39.0 [27.2–39.0.2.0] m; 2w: 51.7 [46.1–54.7.1.7] m; sham: 48.0 [42.2–51.5.2.5] m) or in the time in the center zone among groups (*p* = 0.4211, 1d: 0.94 [0–2.36.36] s; 2d: 0.98 [0.13–3.13.13.13] s; 1w: 0.27 [0–2.03.03] s; 2w: 2.50 [1.35–7.11.35.11] s; sham: 1.29 [0.11–2.73.11.73] s) (Supplementary Fig. S4). Similarly, for the FST, no significant differences were observed in total distance moved (*p* = 0.1475, 1d: 2111.7 ± 406.2 cm; 2d: 1927.8 ± 497.6 cm; 1w: 1957.9 ± 351.1 cm; 2w: 1612.5 ± 272.8 cm; sham: 2003.1 ± 249.0 cm) or in immobile time (*p* = 0.9999, 1d: 155.6 ± 58.8 s; 2d: 152.1 ± 59.3 s; 1w: 156.3 ± 71.0 s; 2w: 153.0 ± 29.4 s; sham: 153.1 ± 50.9 s) (Supplementary Fig. S5) among groups.

### Histological findings

#### Short-interval concussions induced an accumulation of Iba-1-positive microglia in the CC, DG, and CA1

**Fig. 3:**
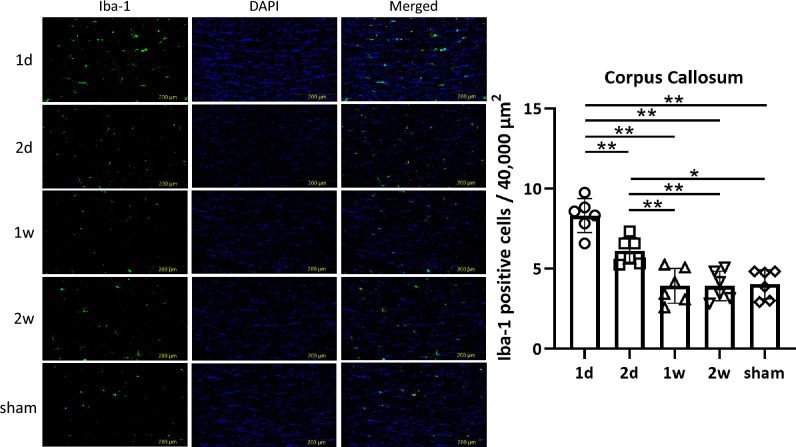
Microglia in the CC. In the CC, one-way ANOVA revealed a significant difference in the total number of microglia among groups (*p* < 0.0001). In addition, unpaired *t*-test indicated a marked reduction in microglia in the 1w, 2w, and sham groups (1d vs. 2 d; *p* = 0.0026, 1 d vs. 1w; *p* < 0.0001, 1 d vs. 2w; *p* < 0.0001, 1 d vs. sham; *p* < 0.0001, 2 d vs. 1w; *p* = 0.0029, 2 d vs. 2w; *p* = 0.0014, 2 d vs. sham; *p* = 0.0020, 1w vs. 2w; *p* = 0.9814, 1w vs. sham; *p* = 0.8628, 2w vs. sham; *p* = 0.8298, respectively) (**p < 0.01). Moreover, an increase in Iba-1 positive microglia exhibiting morphological features of activation in the 1 d group was also observed.

**Fig. 4:**
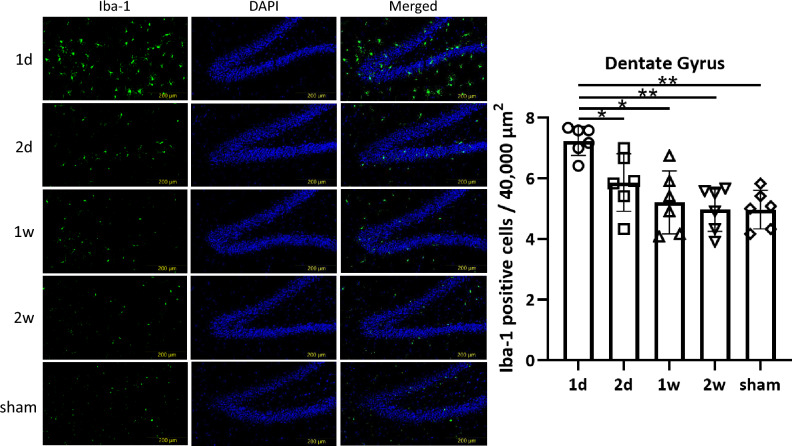
Microglia in the DG. In the DG, one-way ANOVA revealed a significant difference in the total number of microglia among groups (*p* = 0.0001). In addition, unpaired *t*-test showed a marked reduction in microglia in the 1w, 2w, and sham groups (1d vs. 2 d; *p* = 0.0100, 1 d vs. 1w; *p* = 0.014, 1 d vs. 2w; *p* < 0.0001, 1 d vs. sham; *p* < 0.0001, 2 d vs. 1w; *p* = 0.2809, 2 d vs. 2w; *p* = 0.1031, 2 d vs. sham; *p* = 0.0893, 1w vs. 2w; *p* = 0.6764, 1w vs. sham; *p* = 0.6460, 2w vs. sham; *p* = 0.9726, respectively) (*p < 0.05, **p < 0.01). Moreover, an increase in Iba-1 positive microglia exhibiting morphological features of activation in the 1 d group was also observed.

**Fig. 5:**
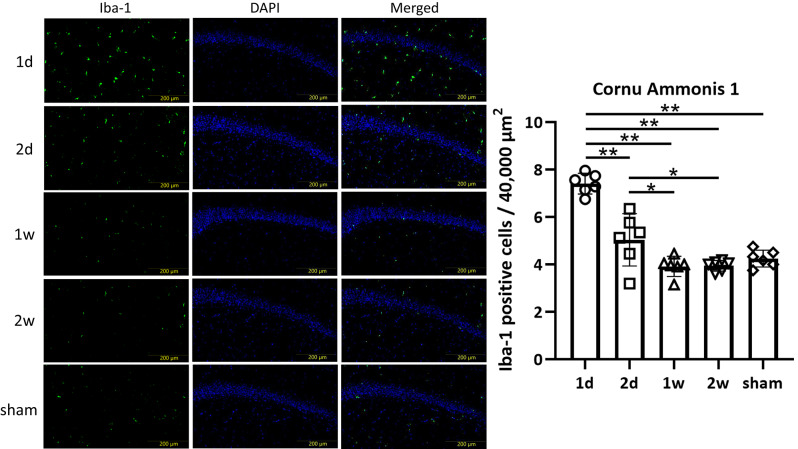
Microglia in the CA1. In the CA1, there were significant differences in the total number of microglia among groups (*p* < 0.0001). In addition, unpaired *t*-test revealed a marked reduction of microglia in 1w, 2w, and sham groups (1d vs. 2 d; *p* < 0.0001, 1 d vs. 1w; *p* < 0.0001, 1 d vs. 2w; *p* < 0.0001, 1 d vs. sham; *p* < 0.0001, 2 d vs. 1w; *p* = 0.0430, 2 d vs. 2w; *p* = 0.0384, 2 d vs. sham; *p* = 0.1456, 1w vs. 2w; *p* = 0.9113, 1w vs. sham; *p* = 0.1803, 2w vs. sham; *p* = 0.1136, respectively) (*p < 0.05, **p < 0.01). Moreover, an increase in Iba-1 positive microglia exhibiting morphological features of activation in the 1 d group was also observed.

Iba-1 staining revealed that the total number of Iba-1 positive cells was significantly higher in the 1 d group compared to other groups in the CC (*p* < 0.0001, 1d: 8.31 ± 0.97; 2d: 6.13 ± 0.76; 1w: 3.93 ± 0.99; 2w: 3.91 ± 0.84; sham: 4.03 ± 0.83), DG (*p* = 0.0001, 1d: 7.24 ± 0.44; 2d: 5.86 ± 0.86; 1w: 5.21 ± 0.94; 2w: 4.99 ± 0.67; sham: 4.97 ± 0.58), and CA1 (*p* < 0.0001, 1d: 7.41 ± 0.40; 2d: 5.04 ± 1.01; 1w: 3.92 ± 0.39; 2w: 3.95 ± 0.21; sham: 4.25 ± 0.33), but not in the PFC (*p* = 0.0697, 1d: 12.1 ± 1.27; 2d: 11.0 ± 0.47; 1w: 11.9 ± 0.86; 2w: 11.0 ± 0.34; sham: 10.9 ± 0.61) (Fig. 3, 4, 5, Supplementary Fig. S6).

Additionally, semiquantitative analysis revealed an increase in Iba-1 positive microglia exhibiting morphological features of activation in the 1 d group in the CC (1d: 3+; 2d: 2+; 1w: 1+; 2w: 1+), DG (1d: 3+; 2d: 2+; 1w: 1+; 2w: 1+), and CA1 (1d: 3+; 2d: 2+; 1w: 1+; 2w: 1+). Using the same scoring criteria, the PFC also showed higher proportion of Iba-1 positive microglia exhibiting morphological features of activation (1d: 3+; 2d: 3+; 1w: 3+; 2w: 3+), but no significant differences were observed among groups (Table 2). These results suggest that short-interval concussions promote an accumulation of microglia in the CC, DG, and CA1, but not in the PFC.

**Table 2:** Semiquantitative analysis of number of Iba-1 positive microglia exhibiting morphological features of activation.

	CC	PFC	DG	CA1
1d	3+	3+	3+	3+
2d	2+	3+	2+	2+
1w	1+	3+	1+	1+
2w	1+	3+	1+	1+

The number of Iba-1 positive microglia exhibiting morphological features of activation was scored as 1+, 2+, or 3+ to indicate 0–1 cells, 2–3 cells, and more than 4 cells per 40,000 µm^2^, respectively.

#### Astrocytes were significantly increased in the PFC, DG, and CA1 with shortened time intervals between concussions

**Fig. 6:**
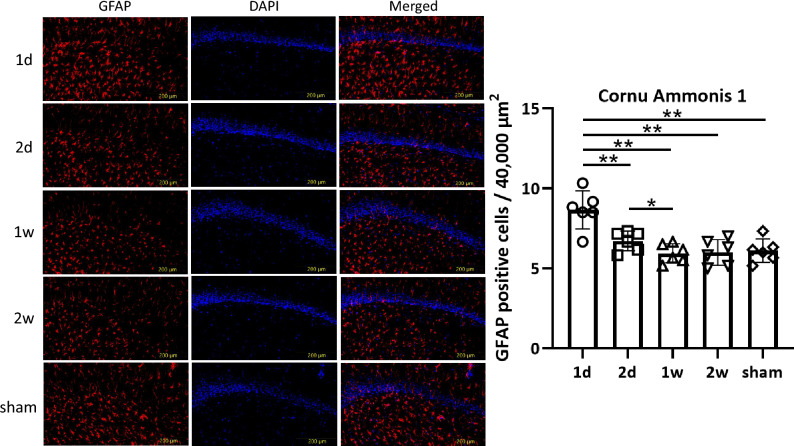
Astrocytes in the CA1. In the CA1, one-way ANOVA revealed significant differences in the number of astrocytes among groups (*p* < 0.0001). Unpaired *t*-test showed a marked reduction in astrocytes in the 2 d, 1w, 2w, and sham groups (1d vs. 2 d; *p* = 0.0052, 1 d vs. 1w; *p* = 0.0005, 1 d vs. 2w; *p* = 0.0011, 1 d vs. sham; *p* = 0.0019, 2 d vs. 1w; *p* = 0.0493, 2 d vs. 2w; *p* = 0.1119, 2 d vs. sham; *p* = 0.1473, 1w vs. 2w; *p* = 0.8948,, 1w vs. sham; *p* = 0.6733, 2w vs. sham; *p* = 0.8077, respectively) (*p < 0.05, **p < 0.01).

GFAP staining revealed that the number of GFAP positive cells was significantly higher in the 1 d group compared to other groups in the PFC (*p* < 0.0001, 1d: 14.1 ± 1.29; 2d: 14.1 ± 1.29; 1w: 10.9 ± 1.02; 2w: 10.3 ± 0.55; sham: 10.2 ± 0.69), DG (*p* < 0.0001, 1d: 14.3 ± 1.07; 2d: 10.8 ± 0.53; 1w: 8.53 ± 0.28; 2w: 8.67 ± 0.55; sham: 8.50 ± 0.43), and CA1 (*p* < 0.0001, 1d: 8.67 ± 1.09; 2d: 6.72 ± 0.56; 1w: 5.94 ± 0.54; 2w: 6.00 ± 0.74; sham: 6.11 ± 0.67), but not in the CC (*p* = 0.5466, 1d: 2.42 [2.35–2.42.35.42]; 2d: 2.38 [2.33–2.48.33.48]; 1w: 2.42 [2.23–2.67.23.67]; 2w: 2.12 [1.79–2.54.79.54]; sham: 2.30 [2.19–2.40.19.40]) (Fig. 6, Supplementary Fig. S7, 8, 9). These results suggest that short-interval concussions promote astrogliosis in the PFC, DG, and CA1, but not in the CC.

#### p-tau accumulation was significantly higher in the PFC and CA1 with shortened time intervals, while minimal accumulation was observed in the CC and DG

In addition, p-tau accumulation was significantly higher in the PFC (*p* < 0.0001, 1d: 1286.8 ± 200.0; 2d: 943.0 ± 133.2; 1w: 863.4 ± 98.2; 2w: 874.0 ± 136.7; sham: 839.9 ± 77.9) and CA1 (*p* = 0.0063, 1d: 58.2 [55.5–62.8.5.8]; 2d: 13.6 [13.1–20.0.1.0]; 1w: 13.9 [11.4–19.1.4.1]; 2w: 13.7 [13.1–17.2.1.2]; sham: 16.4 [14.3–19.2.3.2]), but not in the CC and DG (Supplementary Fig. S10, 11). In both the CC and DG, minimal p-tau accumulation was observed across all groups. These results suggest that short-interval concussions promote p-tau accumulation in the PFC and CA1.

### Discussion

This study evaluated the relationship between the time interval of concussions and changes in behavior and histology. Our results revealed that cognitive function was worsened by short-interval concussions, whereas motor and anxiety-like behaviors were not affected. Several studies on repetitive concussions support these findings^20,36,37^. Meehan et al. investigated the relationship between concussion intervals and cognitive function in mice using a weight-drop model^20^. Mice received five concussions either daily, weekly, or monthly. In their study, mice subjected to daily and weekly concussions exhibited cognitive impairment in the Morris water maze (MWM) test 1 month after the final concussion. Notably, mice in the daily concussion group continued to display cognitive impairment even 1 year after the final concussion. The authors concluded that repetitive concussions delivered within a vulnerable time window may cause long-term, potentially permanent, cognitive impairment in mice. They also suggested that the detrimental effects of repetitive concussions are not solely due to the cumulative influence of injuries, but are also influenced by the time between injuries.

Similarly, Mannix et al. used a weight-drop model to explore repetitive concussions in mice (5–10 impacts in total) and found that those receiving daily or weekly concussions exhibited persistent cognitive impairment in the MWM test up to 1 year post-concussions, while mice concussed every 2 weeks or monthly did not show such impairments^36^. Another study showed that five mTBIs delivered every 2 days led to worsened NORT scores in mice 3 weeks after mTBI, indicating that repetitive mTBI exacerbates cognitive function^37^.

Overall, extending the time interval between concussions appears to reduce the risk of cognitive impairment. This finding aligns with other reports investigating cognitive impairments caused by repetitive concussions^38–40^. Extending the time between concussions may allow athletes to mitigate the cumulative effects of repetitive concussions.

In our study, the number of Iba-1-positive microglia was increased in the 1 d group in the CC, DG, and CA1. In contrast, astrogliosis was observed in the 1 d group in the PFC, DG, and CA1. According to previous reports, the CC, PFC, and DG are among the most frequently reported areas affected after mTBI^41^. In addition to these three regions, we evaluated the CA1 region of the hippocampus, which is known to play essential roles in memory and cognition^42^. CA1 activity is increased following concussion during periods of prolonged inactivity^43^. The number of Iba-1 and GFAP positive cells in 1w and 2w groups was similar to that in the sham group across all regions. These results suggest that extending the time interval would relieve and limit the microgliosis and astrogliosis.

Several tools exist for evaluating concussion symptoms^13–15^. These tools guide the steps to avoid overlooking a concussion. In addition, the 6-Step Return to Play progression is now widely used as reference to determine when an athlete can return to play. Step 1: return to regular activities, Step 2: light aerobic activity, Step 3: moderate activity, Step 4: heavy, non-contact activity, Step 5: practice with full contact, Step 6: competition. This protocol advises that athletes should only progress to the next step if they remain symptom-free at their current step. Similarly, the National Football League concussion protocol recommends comparable steps, but does not specify the exact duration that a player should be out following a concussion. Returning to athletic activity immediately after a symptom-free period may not be sufficient, as metabolic dysfunction may still be present, increasing vulnerability to cumulative injury. On the other hand, recommending strict rest until complete resolution of symptoms may not be the most effective approach. Relative rest—including normal activities of daily living and limiting screen time—has been shown to be beneficial during the first 2 days after concussion^44^. While some reports recommend rest, others suggest that light activity soon after a concussion may support faster recovery^45^. As part of concussion management, reducing screen use during the first 48 h after concussion is recommended, although this intervention may not be effective beyond that period^46^.

Iba-1 staining has been used to evaluate microglia^47,48^. Microglia account for 5 to 20% of all glial cells and serve as immune cells in the central nervous system^49^. Inflammation in the central nervous system plays a critical role in acute protection against infection and injury^50^. In addition, microglia are essential for clearing debris, promoting the reorganization of neuronal circuits, and supporting repair after damage^51,52^. Acute microglial activation increases brain-derived neurotropic factor and insulin-like growth factor-1^53^. Although microglial activation may be associated with cognitive impairment, it also potentially contributes to brain repair and recovery of cognitive function^54^. Microglial activation increases over the week following repeated mTBI, and persists in some brain regions^55^. In our study, an accumulation of microglia was observed in the 1 d group in the CC, DG, and CA1, which may be associated with cognitive impairment. These findings suggest a close relationship between the time interval of repetitive concussions and microgliosis. Although the number of Iba-1-positive microglia in the PFC was high, this may be influenced by the baseline number of total microglia in the PFC, as the number of Iba-1 positive microglia exhibiting morphological features of activation was similar across groups. Extending the time interval appears to limit the increase in these morphologically activated microglia and may help prevent cognitive impairment.

Repetitive TBI also causes persistent axon injury and microglial reactivity^55^, however, acute reduction of microglia using cluster of differentiation 11b thymidine kinase (CD11b-TK) did not alter the extent of axon injury in mice. Therefore, microglial activation may not be associated with axon injury, and directly targeting axonal injury may be more effective than solely focusing on alleviating the microglial activation^56^.

GFAP staining has been used to observe astrogliosis in numerous studies^57,58^. Astrocytes which activated after TBI has both neuroprotective and neurotoxic effects in the brain and can contribute to astrocyte-related neurodegenerative metabolic changes such as CTE^59^. Repetitive mTBI induces astrogliosis localized in the medium and deep layers of the cortex beneath the impact site. Importantly, the peak of astrocyte activation differs from that of microgliosis. According to the report of Mouzon et al, mice subjected to mTBI (5 times every 2 days) exhibited marked astrogliosis in the cortex beneath the impact site, peaking between 10 and 14 days after mTBI^37^. This suggests that astrocyte activation may occur later than microglial activation. In a similar closed head repeated mTBI model, no differences in the number of Iba-1 positive cells in the CC or hippocampus were found 6 months after concussions in male mice, whereas the number of GFAP positive cells remained elevated^36^. This supports the idea that GFAP positive cells may serve as a biomarker for repetitive mTBI. Another study showed that triple head injuries induced significant astrogliosis in the PFC on day 7 and 1 month later, although no such effect was observed in the DG^60^. Interestingly, we observed the histological changes were worsened with short-interval concussions. However, these histological changes did not fully correspond with the behavioral outcomes. Specifically, 2 d group showed an increase in Iba-1 and GFAP positive cells in some regions, yet they did not exhibit any cognitive impairment. This suggests that histological changes do not always correlate with behavioral impairment. Short interval may not allow sufficient time for recovery of inflammatory responses, resulting in cumulative microgliosis and astrogliosis, whereas long interval may provide sufficient time for recovery. It is possible that the histological changes in 2 d group reflect the recovery process rather than pathological damage, and the histological changes observed in 2-day interval may not be sufficient to exceed the threshold to induce cognitive impairment. This discrepancy highlights the difficulty of fully linking histological changes to behavioral impairment. Additional behavioral assessments and longitudinal analyses are needed to clarify the roles of microglia and astrocytes in the brain’s response to injury.

One of the pathological features of CTE is reactive astrogliosis^61^. Neuroinflammation induces the pathogenesis and accumulation of amyloid-beta (Aβ) and tau^62^. Another study also suggests that repetitive mTBI increases Aβ levels, with this effect dependent on the injury interval^63^. TBI is also associated with an increased risk of Parkinson’s disease. Therefore, extending the time interval between concussions may help to relieve neuroinflammation and limit the accumulation of Aβ and tau, thereby reducing the risk of future neurodegenerative diseases such as CTE or Parkinson’s disease. In our study, significant astrogliosis was observed only in the 1 d group in the PFC, DG, and CA1, suggesting that repetitive concussions may influence the number of astrocytes in specific brain regions. In mice, inhibiting proinflammatory cytokines after TBI has been shown to reduce neurological impairment^64^.

Tau protein exists in axons and plays a role in stabilizing microtubules. Accumulation of excessive tau protein leads to the degeneration of axons and is implicated in Alzheimer’s disease and other tauopathies^65^. Repetitive concussions are known to cause CTE, which is characterized by the accumulation of p-tau^22^. A definitive diagnosis of CTE can only be made by postmortem pathological examination^66^. Petraglia et al. reported significant increases in p-tau at 7 days, 1 month, and 6 months post-injury in the cortex, amygdala, and hippocampus following highly repetitive impacts (42 times over 7 days)^67^. Similarly, we previously observed increased accumulation of p-tau 1 month after repetitive concussions (3 times in 3 days) compared to levels seen 2 weeks post-injury^21^. These findings suggest that the frequency of concussions is closely related to the accumulation of p-tau, which may gradually increase over time. Accumulation of p-tau following TBI has been associated with worsened behavioral and clinical symptoms, persisting for up to 6 months after injury^68^.

The pathological accumulation of tau protein is not always limited to the site or hemisphere receiving the concussion—it can extend across brain hemispheres over time^35^. One study investigating *in vivo* tau aggregation using positron emission tomography (PET)/magnetic resonance imaging reported that repeated sports-related concussions led to tau aggregation in the hippocampus and CC, changes not observed in healthy controls. In addition, increased tau aggregation and neuroinflammation were observed more than 6 months post-concussion using PET imaging, although it remains unclear whether these changes inevitably lead to CTE in the future^66^.

In our study, p-tau accumulation was significant in the PFC and CA1, consistent with previous reports^21,35^. However, a separate mouse model of repetitive concussion using weight drops (5–10 impacts in total) revealed that long-term cognitive impairment was associated with increased astrocytes but not p-tau or Aβ accumulation^36^. These findings suggest that the contribution of p-tau accumulation to cognitive impairment may differ between rats and mice.

The accumulation of tau protein is a marker of neurodegeneration and provides valuable insight into the long-term effects of TBI. In summary, our study revealed significant p-tau accumulation in the PFC and CA1. These findings support a relationship between the time interval of repetitive concussions and region-specific p-tau accumulation in the brain, which may signal an increased risk of neurodegeneration in the future.

In this study, cognitive impairment was observed only in the 1 d group, while the 2 d group did not exhibit such impairment. Therefore, a 2-day interval appears to significantly reduce the risk of long-lasting cognitive impairment, suggesting that the threshold to prevent cognitive impairment following repetitive concussions lies between 1 and 2 days. Importantly, the definition of “1 day” differs between rats and humans. A single day for a rat is equivalent to approximately 27 days for a human^67^. Another study highlights the importance of considering the development stage of rats. Five-week-old rats are in their adolescent period, during which 10.5 rat-days are equivalent to 1 human year. That is to say, a single day for adolescent rats is roughly equivalent to 34.8 days for humans^68^. Based on these findings, we infer that a 30- to 60-day interval between concussions may reduce the risk of cognitive impairment in humans.

However, the severity of trauma also influences brain function. Although the force of trauma was assessed in this study, no research has precisely measured the force of concussion in humans due to ethical constraints. Consequently, it remains unclear how the traumatic force applied in rats compared to that experienced by humans. Further studies are needed to investigate both the time interval and the force of concussion. Depending on the force of the concussion, a longer interval may be necessary to minimize the risk of cognitive impairment.

### Limitations

There are several limitations in this study. First, the study employed rats, and the human brain may respond differently to injury. Differences in brain size and anatomical structures present challenges when extrapolating rodent concussion models to human pathology. Further investigation using non-human primates should be considered. Second, the age at the time of final concussion varied among the groups due to the consistent timing of evaluations following the final concussion in our experimental time course. Rats in the 2w group were much larger than those in the 1 d group at the time of final concussion. The differences in body size may have influenced the effects of concussions in groups receiving concussions with longer intervals between injuries. Third, immunohistological evaluation of Iba-1 alone cannot definitively indicate the microglial activation. Therefore, the observed increase in Iba-1 positive microglia exhibiting morphological features of activation should be interpreted carefully. Fourth, although we measured the impact force for all concussions, it remains unclear how the force applied to rats translates to human concussion forces. Further investigation exploring the impact force of concussions in humans is needed. Fifth, the use of isoflurane in this study may have introduced a neuroprotective effect, potentially mitigating the impact of the concussions.

## Conclusion

We investigated the relationship between the time interval of concussions and changes in behavior and histology. Cognitive impairment was observed only in rats receiving short-interval concussions. This finding suggests that a longer interval between concussions may reduce the risk of cognitive impairment and limit the microgliosis, astrogliosis, and p-tau accumulation following repetitive concussions. These results may help inform guidelines for determining safe return to play timelines in contact sports in humans.

## Supplementary Information

Supplementary Information 1.

Supplementary Information 2.

## Data Availability

The dataset has been developed by the authors and is accessible from the corresponding author on request.
