# Supplementary material for: Long intervals between repetitive concussions reduce risk of cognitive impairment and limit microglial activation, astrogliosis, and tauopathy in adolescent rats
Source: Sci Rep. 2025 Nov 18;15:40522. doi: 10.1038/s41598-025-24376-y (PMC12627510; doi:10.1038/s41598-025-24376-y)
Supplement: Supplementary file 1 — Supplementary Information 1. [file 41598_2025_24376_MOESM1_ESM.pdf]

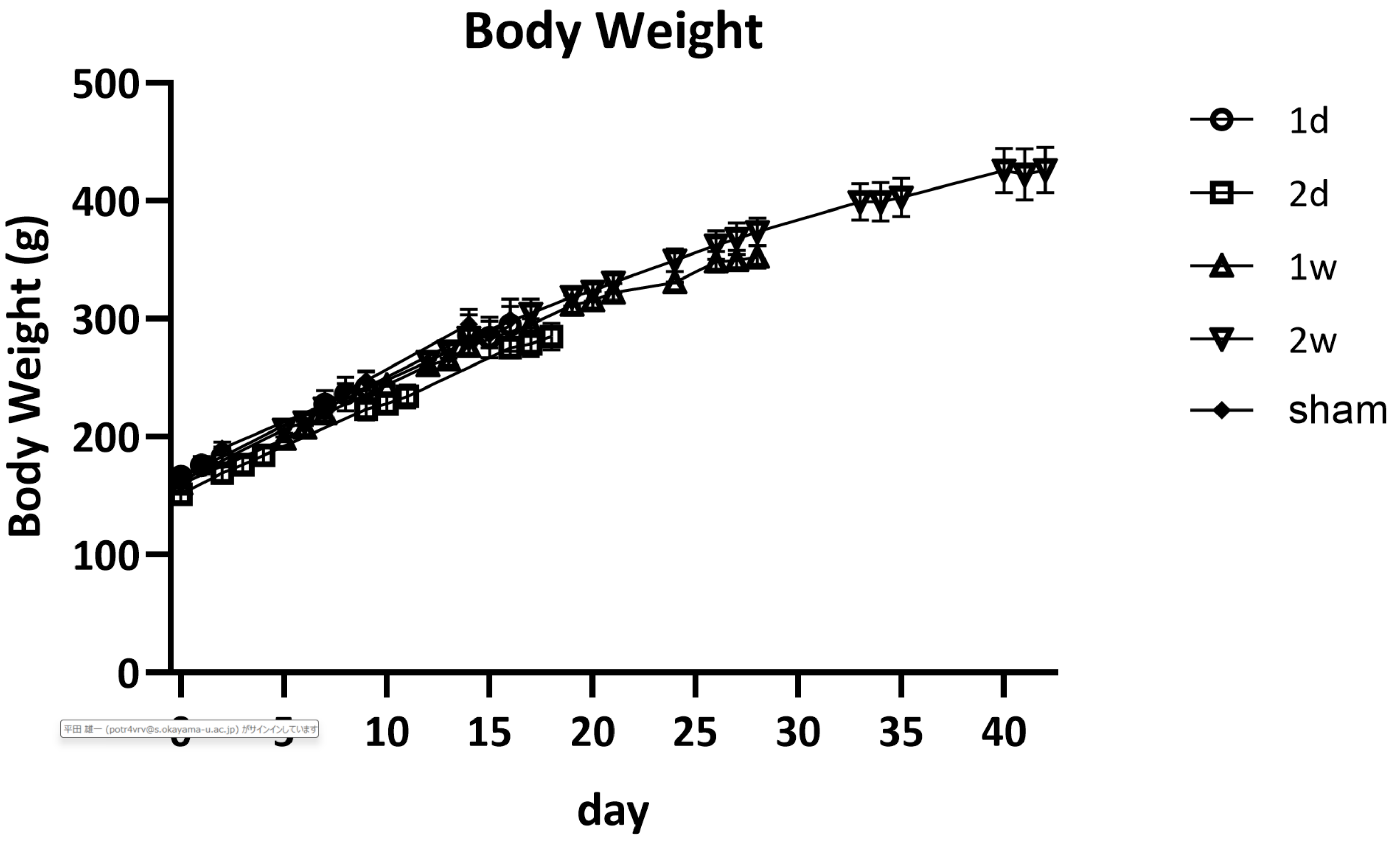

Supplementary Figure S1

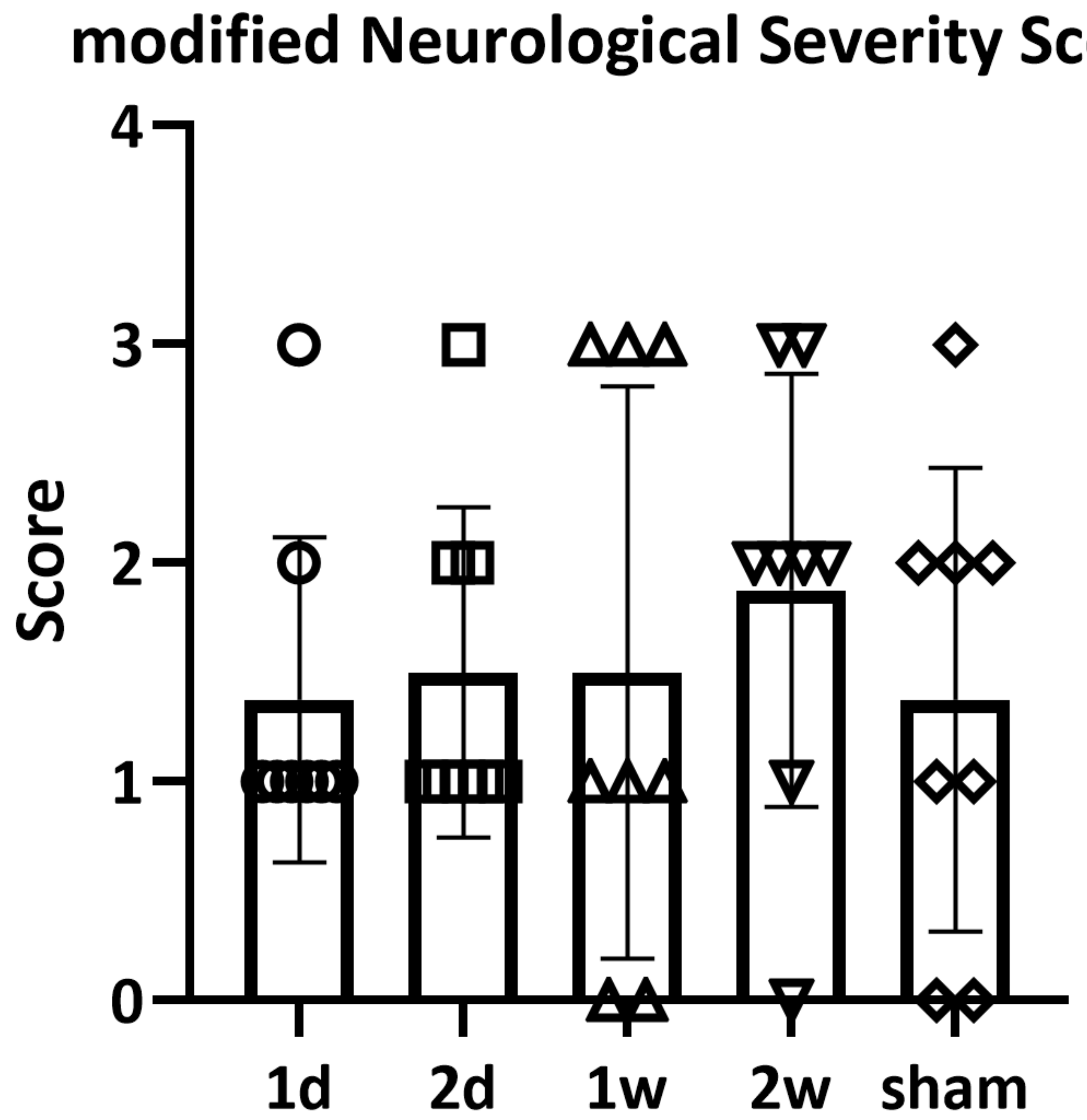

Supplementary Figure S2

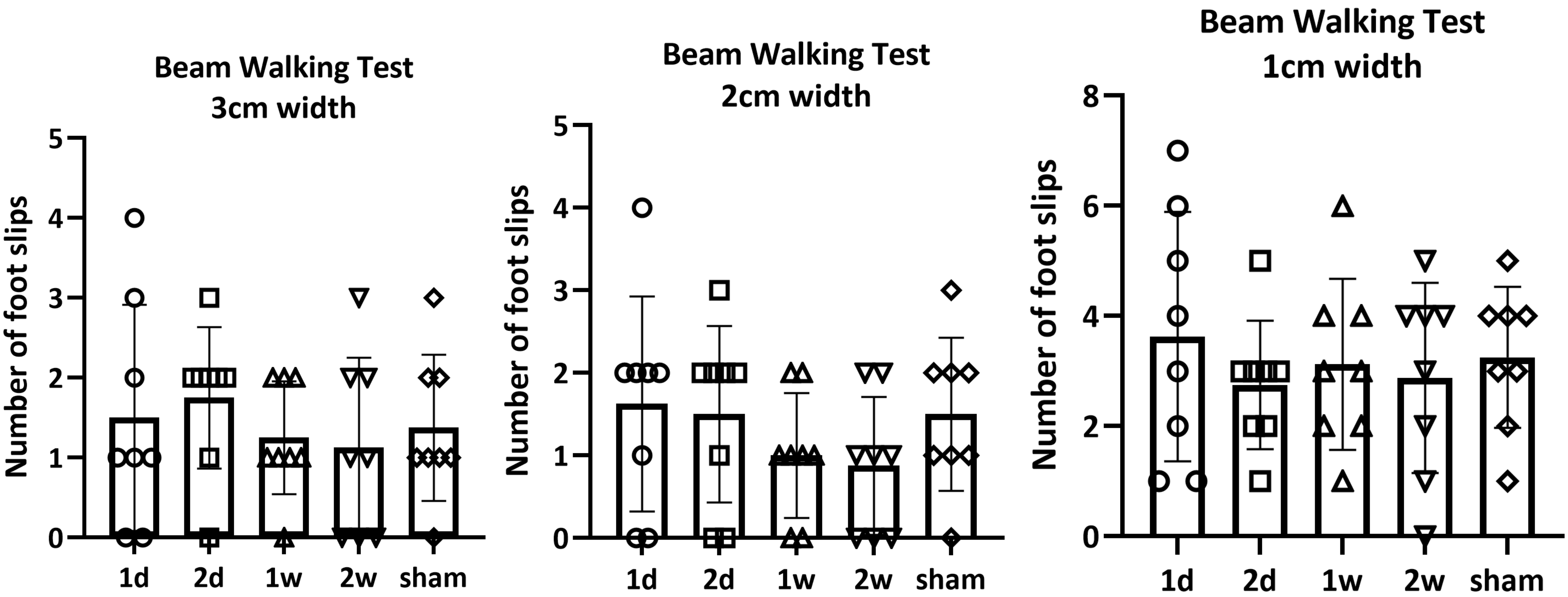

Supplementary Figure S3

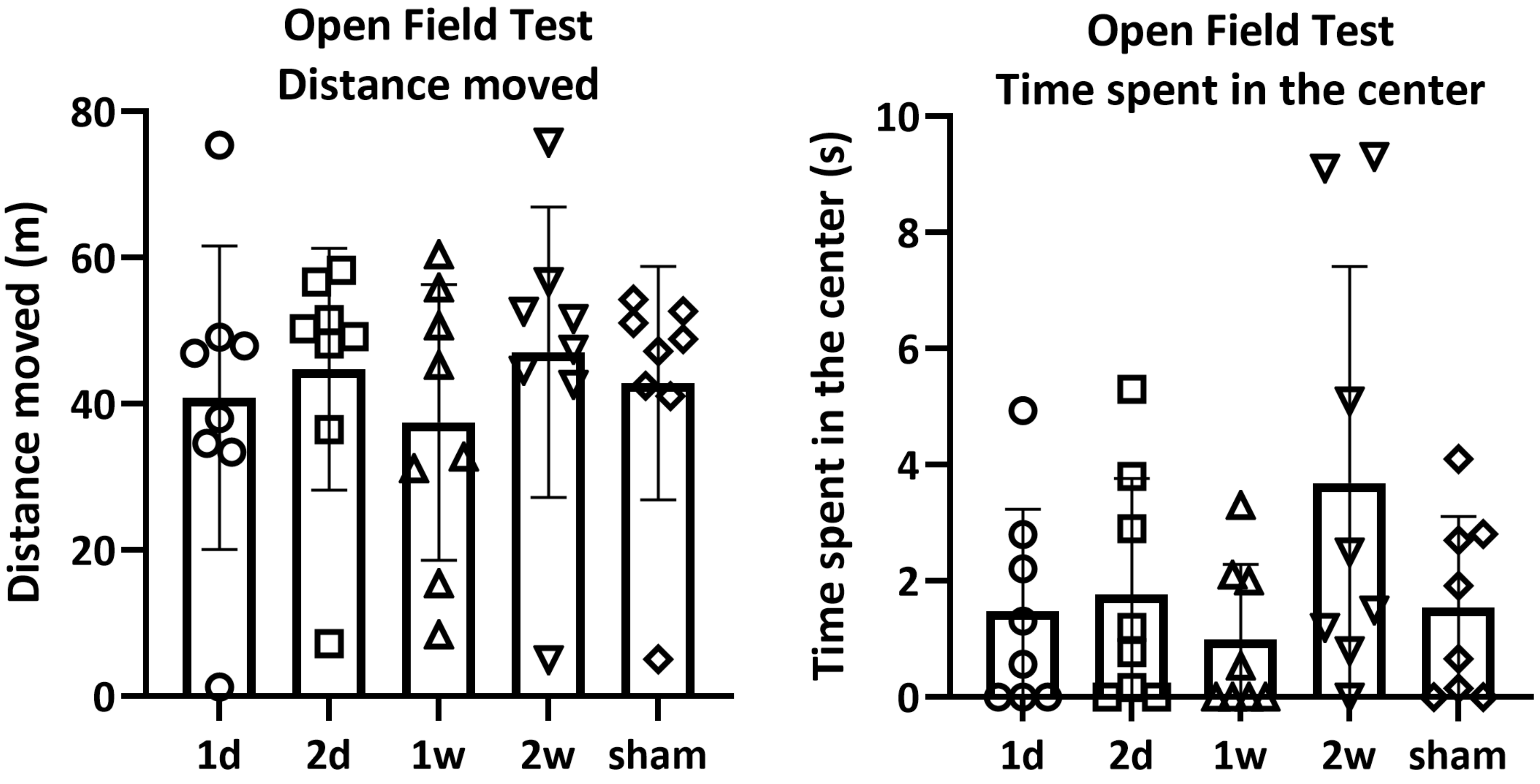

Supplementary Figure S4

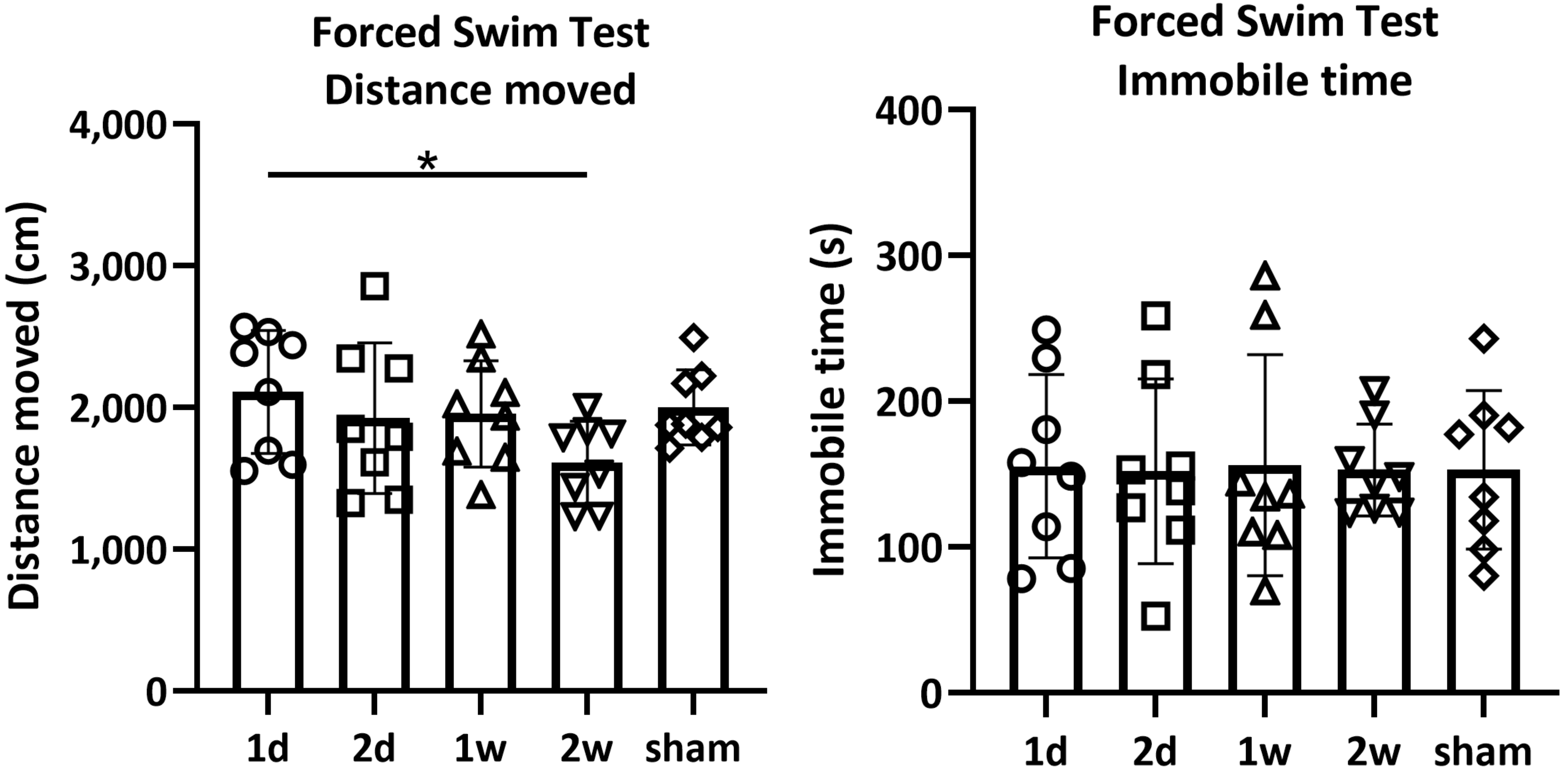

Supplementary Figure S5

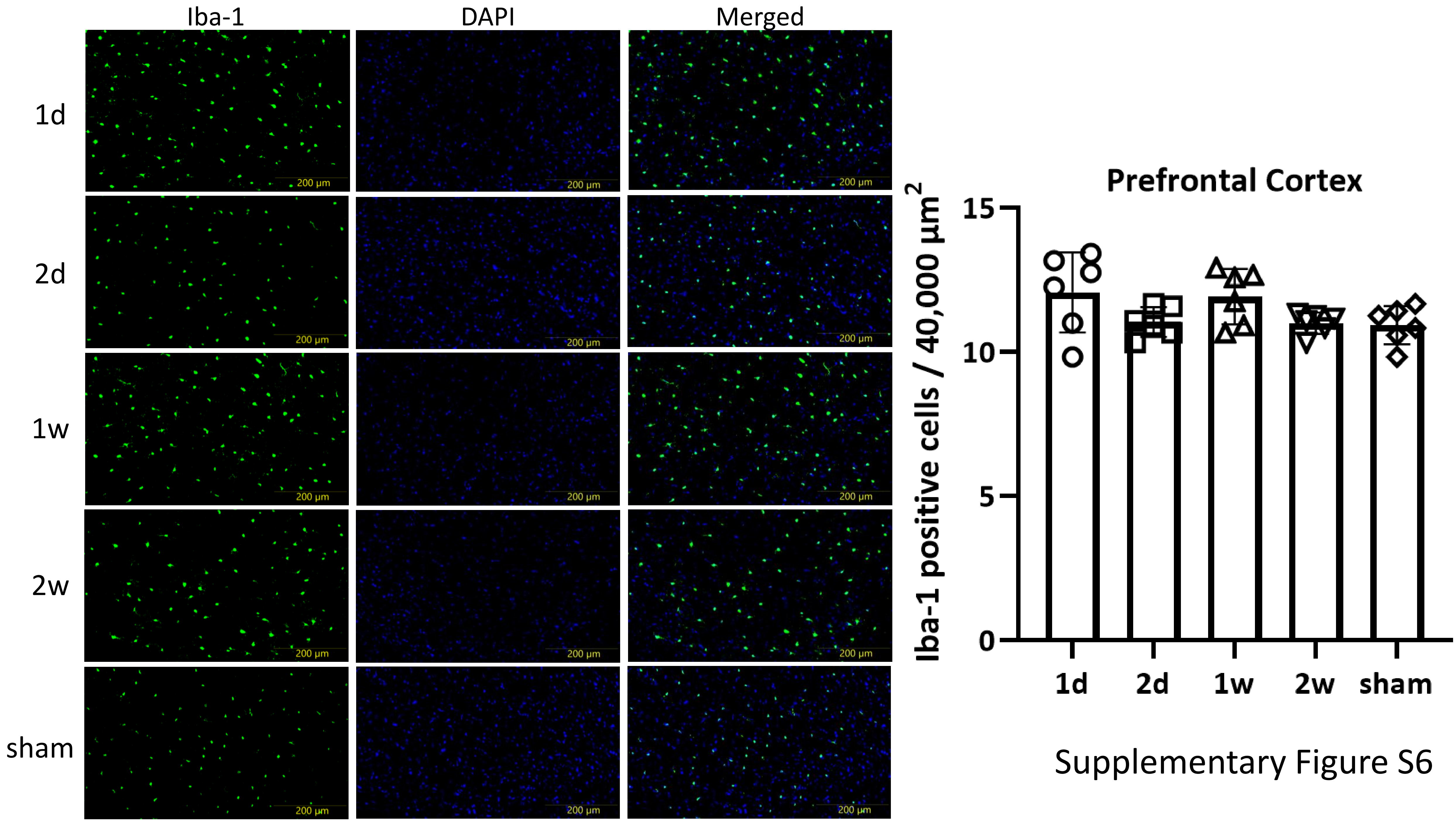

Supplementary Figure S6

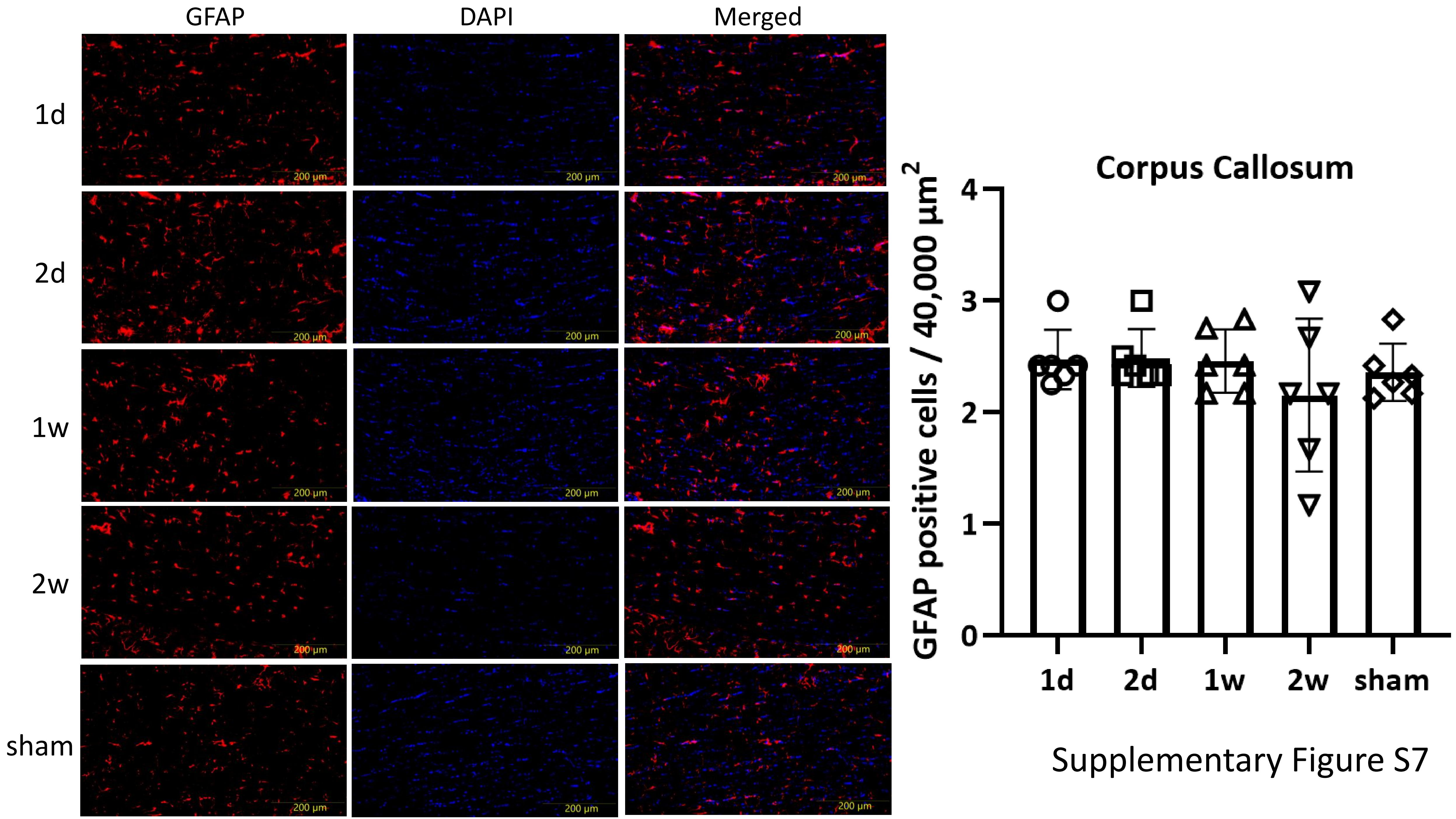

Supplementary Figure S7

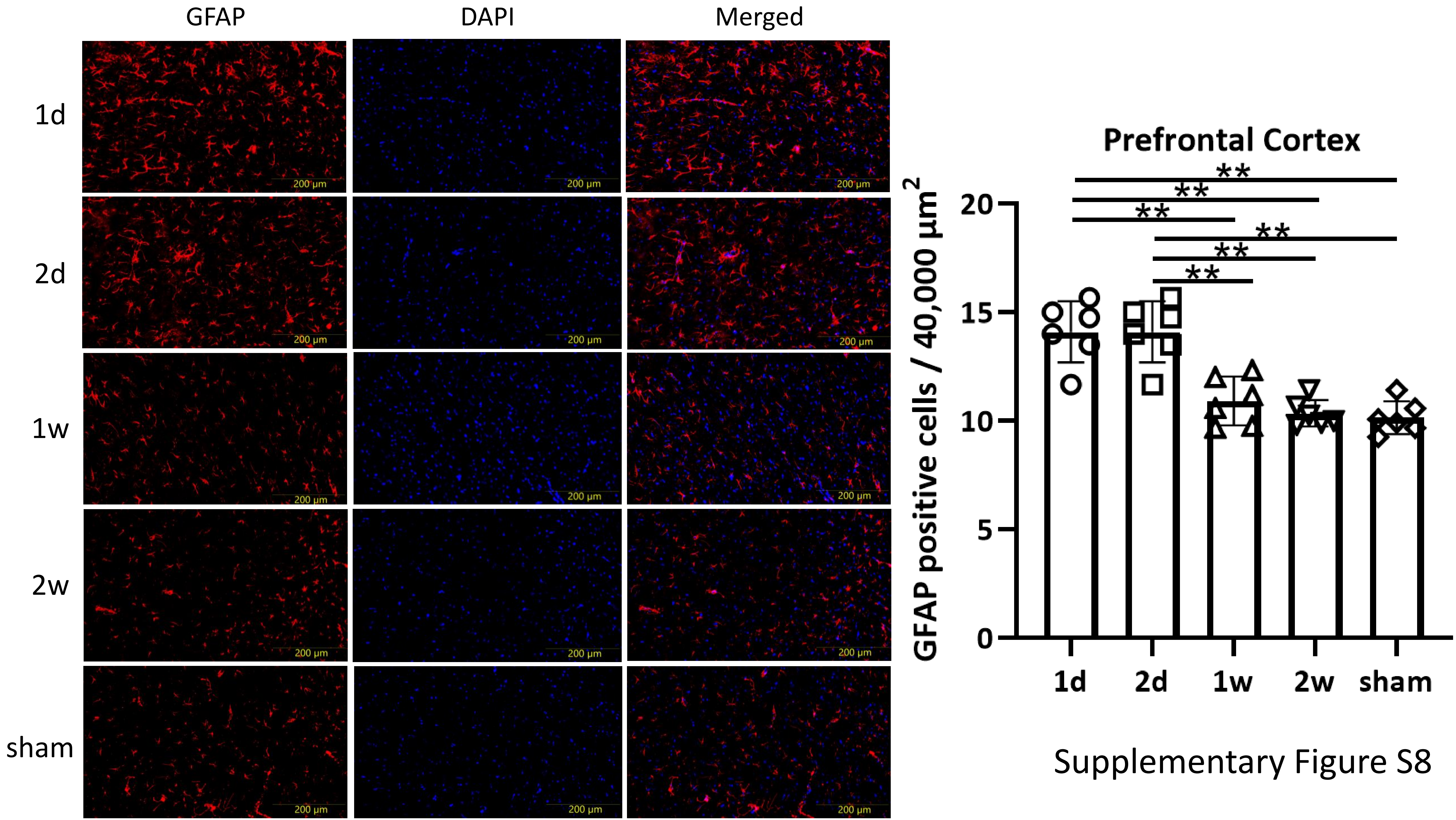

Supplementary Figure S8

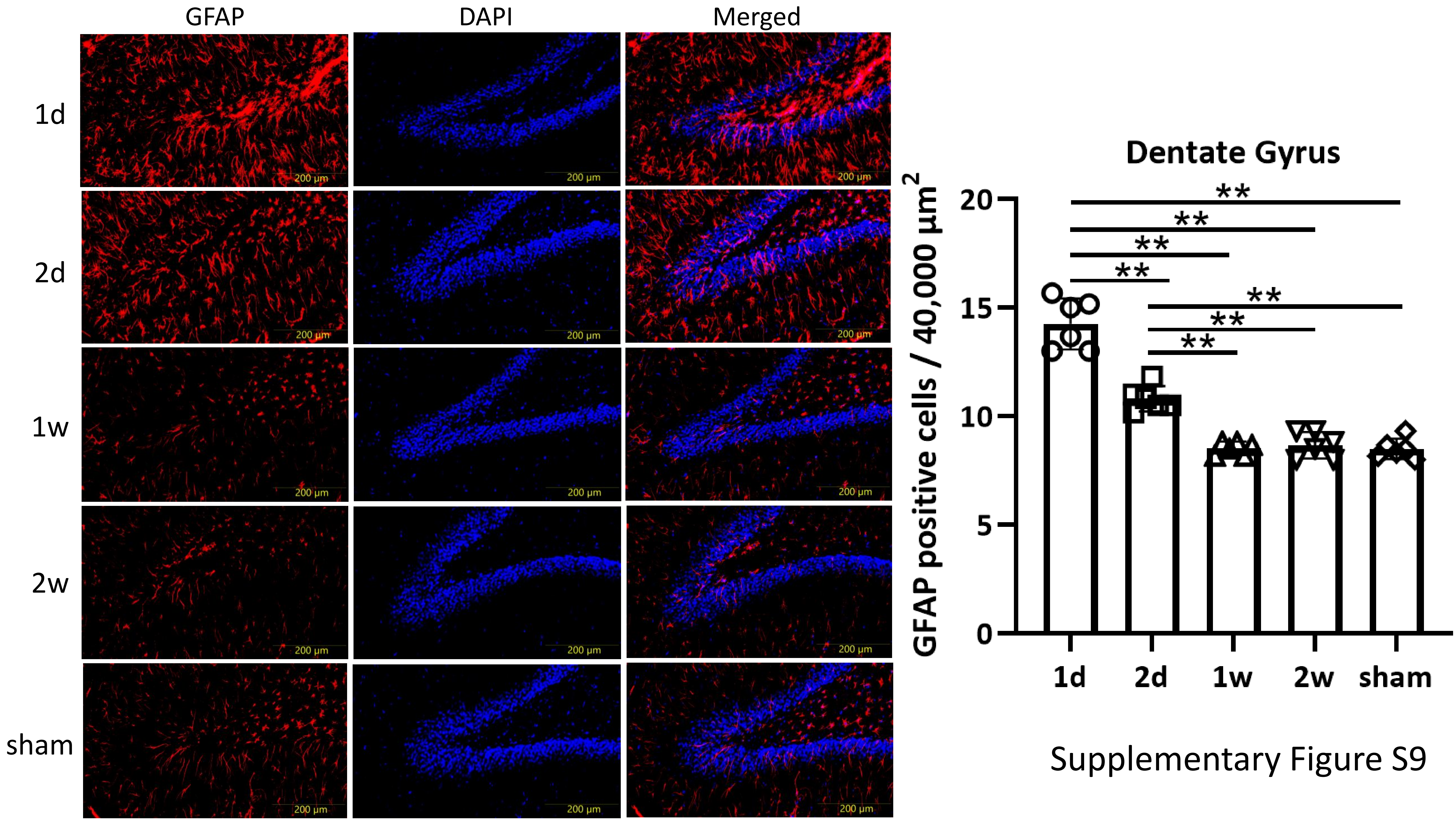

Supplementary Figure S9

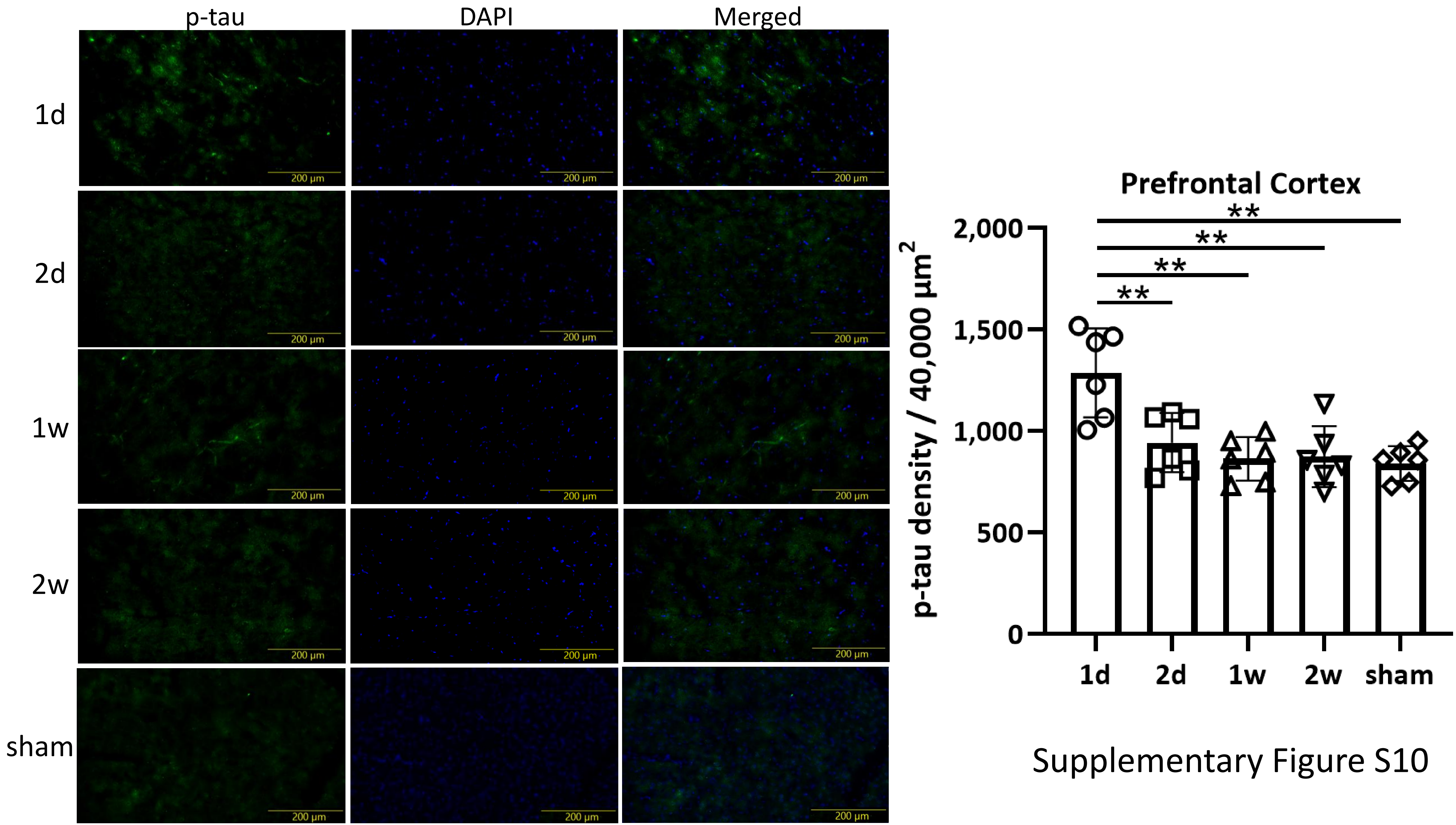

Supplementary Figure S10

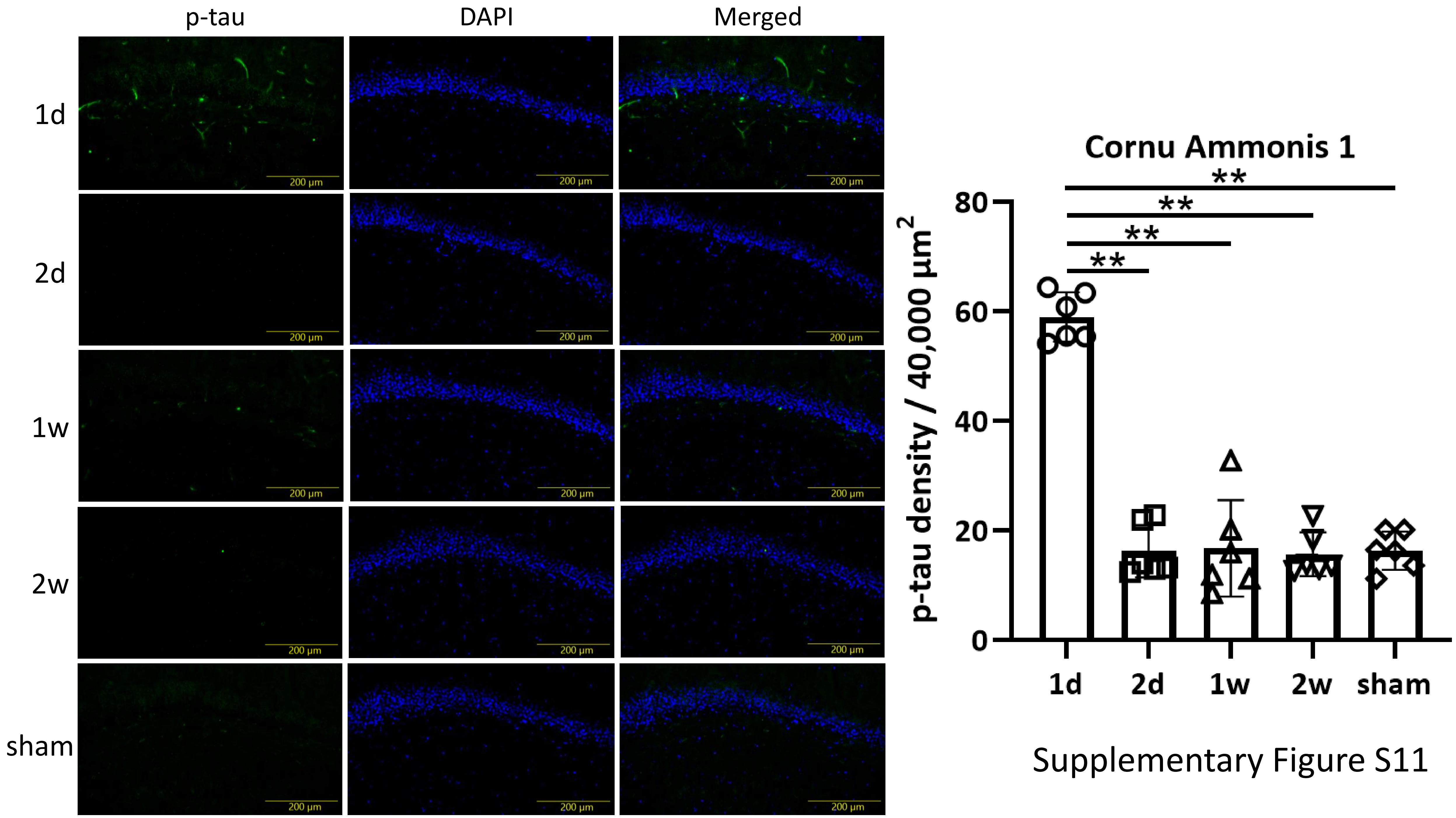

Supplementary Figure S11
