# Supplementary material for: Long intervals between repetitive concussions reduce risk of cognitive impairment and limit microglial activation, astrogliosis, and tauopathy in adolescent rats
Source: Sci Rep. 2025 Nov 18;15:40522. doi: 10.1038/s41598-025-24376-y (PMC12627510; doi:10.1038/s41598-025-24376-y)
Supplement: Supplementary file 2 — Supplementary Information 2. [file 41598_2025_24376_MOESM2_ESM.docx]

**Supplementary Figure legends**

Supplementary Figure S1. Body weight.

Although the euthanasia date varied among groups due to the experimental time course, there was no difference in body weight among groups up to day 14 (mean ± SD, day 0: 1d: 166.4 ± 4.5 g; 2d: 149.1 ± 5.8 g; 1w: 160.5 ± 3.4 g; 2w: 161.0 ± 4.4 g; sham: 166.6 ± 3.6 g, day 14: 1d: 294.8 ± 20.2 g; 2d: 286.8 ± 10.3 g; 1w: 275.0 ± 5.5 g; 2w: 285.8 ± 5.7 g; sham: 299.8 ± 10.0 g).

Supplementary Figure S2. Results of the mNSS.

The Kruskal-Wallis test revealed no significant difference in the mNSS score among groups (*p* = 0.7811, median [IQR], 1d: 1 [1.0-1.3]; 2d: 1 [1.0-2.0]; 1w: 1 [0.8-3.0]; 2w: 2 [1.8-2.3]; sham: 1.5 [0.8-2.0]).

Supplementary Figure S3. Results of the BWT.

One-way ANOVA revealed no significant difference in the number of foot slips in the BWT among groups (3 cm, 2 cm, 1 cm, *p* = 0.7868, *p* = 0.4638, *p* = 0.8445, mean ± SD, 1d: 1.5 ± 1.3, 1.6 ± 1.2, 3.6 ± 2.1; 2d: 1.8 ± 0.8, 1.5 ± 1.0, 2.8 ± 1.1; 1w: 1.3 ± 0.7, 1.0 ± 0.7, 3.1 ± 1.5; 2w: 1.1 ± 1.1, 0.9 ± 0.8, 2.9 ± 1.6; sham: 1.4 ± 0.9, 1.5 ± 0.9, 3.3 ± 1.2).

Supplementary Figure S4. Results of the OFT.

The Kruskal-Wallis test revealed no significant difference in the total distance moved in the arena among groups (*p* = 0.6439, median [IQR], 1d: 42.5 [34.3-48.3] m; 2d: 49.8 [45.3-52.8] m; 1w: 39.0 [27.2-39.0] m; 2w: 51.7 [46.1-54.7] m; sham: 48.0 [42.2-51.5] m). Likewise, no significant difference was found in the time spent in the center (*p* = 0.4211, 1d: 0.94 [0-2.36] s; 2d: 0.98 [0.13-3.13] s; 1w: 0.27 [0-2.03] s; 2w: 2.50 [1.35-7.11] s; sham: 1.29 [0.12-2.73] s).

Supplementary Figure S5. Results of the FST.

One-way ANOVA revealed no significant difference in the total distance moved in the arena among groups (*p* = 0.1475, mean ± SD, 1d: 2111.7 ± 406.2 cm; 2d: 1927.8 ± 497.6 cm; 1w: 1957.9 ± 351.1 cm; 2w: 1612.5 ± 272.8 cm; sham: 2003.1 ± 249.0 cm). Likewise, there was no significant difference in immobility time among groups (*p* = 0.9999, 1d: 155.6 ± 58.8 s; 2d: 152.1 ± 59.3 s; 1w: 156.3 ± 71.0 s; 2w: 153.0 ± 29.4 s; sham: 153.1 ± 50.9 s) (**p* < 0.05).

Supplementary Figure S6. Microglia in the PFC.

In the PFC, one-way ANOVA revealed no significant difference in the total number of microglia (*p* = 0.0697). In addition, the number of Iba-1 positive microglia exhibiting morphological features of activation was similar across all groups.

Supplementary Figure S7. Astrocytes in the CC.

In the CC, the Kruskal-Willis test revealed no significant difference in the number of astrocytes among groups (*p* = 0.5466).

Supplementary Figure S8. Astrocytes in the PFC.

In the PFC, one-way ANOVA revealed a significant difference in the number of astrocytes among groups (*p* < 0.0001). In addition, unpaired *t*-test showed a considerable reduction in astrocytes in the 1w, 2w, and sham groups (1d vs. 2d; *p* > 0.9999, 1d vs. 1w; *p* = 0.0015, 1d vs. 2w; *p* = 0.0001, 1d vs. sham; *p* = 0.0004, 2d vs. 1w; *p* = 0.0015, 2d vs. 2w; *p* = 0.0001, 2d vs. sham; *p* = 0.0004, 1w vs. 2w; *p* = 0.2989, 1w vs. sham; *p* = 0.2012, 2w vs. sham; *p* = 0.6351, respectively) (***p* < 0.01).

Supplementary Figure S9. Astrocytes in the DG.

In the DG, one-way ANOVA revealed a significant difference in the number of astrocytes among groups (*p* < 0.0001). In addition, unpaired t-test showed a marked reduction in astrocytes in the 1w, 2w, and sham groups (1d vs. 2d; *p* < 0.0001, 1d vs. 1w; *p* < 0.0001, 1d vs. 2w; *p* < 0.0001, 1d vs. sham; *p* < 0.0001, 2d vs. 1w; *p* < 0.0001, 2d vs. 2w; *p* < 0.0001, 2d vs. sham; *p* < 0.0001, 1w vs. 2w; *p* = 0.6269, 1w vs. sham; *p* = 0.9064, 2w vs. sham; *p* = 0.6070, respectively) (***p* < 0.01).

Supplementary Figure S10. p-tau in the PFC.

In the PFC, one-way ANOVA revealed significant differences in the accumulation of p-tau among groups (*p* < 0.0001). Additionally, unpaired *t*-test showed a marked reduction in the accumulation of p-tau in the 1w, 2w, and sham groups (1d vs. 2d; *p* = 0.0095, 1d vs. 1w; *p* = 0.0017, 1d vs. 2w; *p* = 0.0034, 1d vs. sham; *p* = 0.0028, 2d vs. 1w; *p* = 0.3072, 2d vs. 2w; *p* = 0.4374, 2d vs. sham; *p* = 0.1731, 1w vs. 2w; *p* = 0.8910, 1w vs. sham; *p* = 0.6841, 2w vs. sham; *p* = 0.6411, respectively) (***p* < 0.01).

Supplementary Figure S11. p-tau in the CA1.

In the CA1, the Kruskal-Willis test revealed significant differences in the accumulation of p-tau among groups (*p* = 0.0063). Additionally, unpaired *t*-test and Mann-Whitney *U*-test showed marked p-tau accumulation in the 1d group compared to the other groups (1d vs. 2d; *p* = 0.0022, 1d vs. 1w; *p* < 0.0001, 1d vs. 2w; *p* = 0.0022, 1d vs. sham; *p* < 0.0001, 2d vs. 1w; *p* = 0.5887, 2d vs. 2w; *p* = 0.9740, 2d vs. sham; *p* = 0.9004, 1w vs. 2w; *p* = 0.6991, 1w vs. sham; *p* = 0.9142, 2w vs. sham; *p* = 0.7338, respectively) (***p* < 0.01).
